# Infrared Thermography Approach for Effective Shielding Area of Field Smoke Based on Background Subtraction and Transmittance Interpolation

**DOI:** 10.3390/s18051450

**Published:** 2018-05-06

**Authors:** Runze Tang, Tonglai Zhang, Yongpeng Chen, Hao Liang, Bingyang Li, Zunning Zhou

**Affiliations:** State Key Laboratory of Explosion Science and Technology, Beijing Institute of Technology, Beijing 100081, China; tangrz@bit.edu.cn (R.T.); ztlbit@bit.edu.cn (T.Z.); 2120160188@bit.edu.cn (Y.C.); lh101410401@163.com (H.L.); 2220170083@bit.edu.cn (B.L.)

**Keywords:** infrared thermography, infrared sensor, field trial, effective shielding area, background subtraction, transmittance interpolation

## Abstract

Effective shielding area is a crucial indicator for the evaluation of the infrared smoke-obscuring effectiveness on the battlefield. The conventional methods for assessing the shielding area of the smoke screen are time-consuming and labor intensive, in addition to lacking precision. Therefore, an efficient and convincing technique for testing the effective shielding area of the smoke screen has great potential benefits in the smoke screen applications in the field trial. In this study, a thermal infrared sensor with a mid-wavelength infrared (MWIR) range of 3 to 5 μm was first used to capture the target scene images through clear as well as obscuring smoke, at regular intervals. The background subtraction in motion detection was then applied to obtain the contour of the smoke cloud at each frame. The smoke transmittance at each pixel within the smoke contour was interpolated based on the data that was collected from the image. Finally, the smoke effective shielding area was calculated, based on the accumulation of the effective shielding pixel points. One advantage of this approach is that it utilizes only one thermal infrared sensor without any other additional equipment in the field trial, which significantly contributes to the efficiency and its convenience. Experiments have been carried out to demonstrate that this approach can determine the effective shielding area of the field infrared smoke both practically and efficiently.

## 1. Introduction

The use of infrared thermography in civil and military applications has been growing considerably over the last few decades [1,2,3]. As a result of its fast inspection time, high sensitivity, and large spatial resolution, infrared sensors have been widely utilized in the fields of target detection, military reconnaissance, and missile guidance [4]. The evolution of the infrared sensor and its ability to suppress interference, continually accelerate the development of the electro-optical countermeasures. As one of the primary electro-optical countermeasures used throughout this century, the smoke screen is able to release obscurants over a large area so as to absorb and reflect the target infrared radiation, thus degrading the effectiveness of the sophisticated guided missiles [5,6,7,8,9]. Consequently, the study on the effective shielding area of the smoke in the infrared bands has caused widespread interest [10,11,12,13,14].

The effective shielding area of the infrared smoke is an important parameter for evaluating the obscuring performance of the infrared smoke in the field trial. In order to study the infrared extinction characteristics of the smoke screen, infrared sensors are widely applied in practice so as to observe and record the motion of the smoke, which can simulate the battlefield scene [15]. Previous studies have developed a considerable number of methods to detect the smoke screen, such as wavelets, support vector machines, image fusion techniques, color features, and motion detection [16,17,18,19,20]. In addition, Yang et al. [21] proposed a test method of the geometric smoke area, which is based on the number of image pixels. Zhu et al. [22] suggested an approach to predict the smoke shielding area using the diffusion equation. However, because of the factors of the wind speed, air turbulence, and smoke diffusion characteristics, the dispersion of the smoke screen can lead to large differences in obscuring the performance within different parts of the smoke cloud [23]. This causes clear distinctions between the geometric area and the effective shielding area of the smoke screen. Currently, the most commonly used method for obtaining the effective shielding area involves the subjective evaluation through naked eyes, which cannot guarantee precision. Therefore, a universal and convincing method to determine the effective shielding area of the smoke is strongly desired.

In this paper, we have proposed a practical approach in order to measure the effective shielding area of the field infrared smoke, with the help of an infrared sensor. Firstly, the infrared sensor was utilized to capture the video sequence of the target scene, during the field experiment. Secondly, the geometric contour of the dynamic smoke cloud from each image frame was obtained using the motion detection techniques. After this, according to the grayscale smoke transmittance model [24] and the spatial resolution of the infrared sensor, the smoke transmittance of each pixel within the smoke contour was determined by a linear interpolation. Finally, when compared with the input smoke transmittance threshold, the effective shielding area was calculated using the accumulation of the effective shielding pixel points. This approach is capable of measuring the effective shielding area of the smoke in the field trial, with advantages in the economy, convenience, and efficiency.

## 2. Experimental Setup

**Figure 1:**
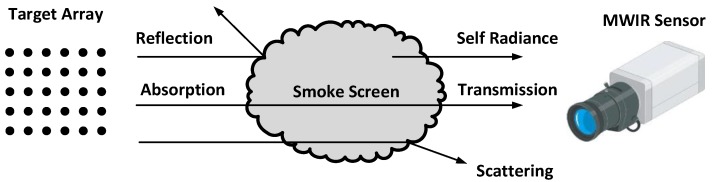
Schematic diagram of the field experiment. MWIR—mid-wavelength infrared.

The experimental setup consisted of three parts, namely: (1) an infrared radiation target array; (2) a smoke release site; and (3) a measurement system site [24]. The test site was chosen in order to get enough distance between the radiation sources and the measurement system. The distance between the targets and the sensor was selected as 500 m in the field trial, in order to obtain the best spatial resolution for the imaging systems. In addition, the weather conditions, especially the wind speed and temperature, could determine the atmospheric stability, which would thus have influenced the results of the observed quantities. Consequently, the experiment was carried out under stable meteorological conditions when the temperature was approximately 10–30 °C and the wind speed was under 5 m/s. The schematic diagram of the field experiment in this study is shown in Figure 1.

The Standard blackbodies (SR-800N Superior Accuracy Blackbody, CI System Ltd., Migdal HaEmek, Israel) that emitted the infrared radiation were fixed in lines and columns vertically, in order to form an infrared signal array. Each blackbody had a radiation surface of 20 cm × 50 cm for the thermal sensor measurements and could maintain the preset temperatures in the range of −25 °C to 100 °C. In this experiment, the temperature of the blackbody was set as 37 °C, so as to simulate the human body temperature. The radiation from the targets could be reflected, scattered, and absorbed by the smoke screen during the smoke diffusion process. The thermography images were collected using a commercial mid-wavelength infrared (MWIR) sensor (SZJ-2 Cooling FPA Thermal Imager, Kunming North Infrared Technology Co. Ltd., Kunming, China). The sensor had a cooled InSb focal plane array detector with a resolution of 320 × 240 pixels, which captured the spectra with a wavelength of 3–5 μm. The field of view (FOV) of the sensor was 11° × 8.25° and the lens focal length was 50 mm. In this study, the approach to calculate the effective shielding area of the field infrared smoke was based on the images that were captured from the MWIR sensor, which could approximately simulate the battlefield scene from the infrared seeker within the range of 3–5 μm.

## 3. Methodology

### 3.1. Background Subtraction

Many of the traditional methods were proposed in a long-term study of the motion detection in a continuous video stream, including background subtraction, frame difference, and optical flow [25]. In this section, the background subtraction was applied to detect and track the motion of the smoke cloud.

**Figure 2:**
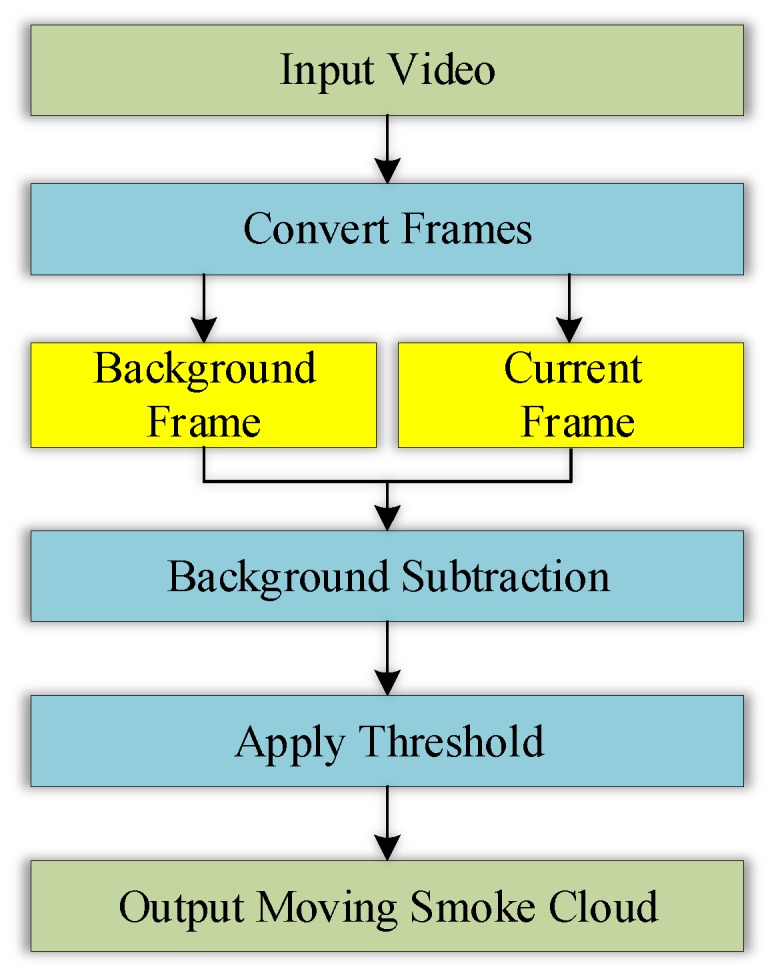
The algorithm flowchart of the background subtraction method.

Background subtraction was a conventional method for detecting moving objects in a static background scene, through a pixel-by-pixel comparison of the current image with a background image, pixel by pixel [26]. The basic idea is expressed in Equation (1) as follows:

(1) $$∆I\left(i,j\right)=I_{1}\left(i,j\right)-I_{0}\left(i,j\right)$$

where ∆I(i,j) is the difference in the image intensity between the current and background image frames, at a certain pixel point (i,j); and $I_{1}\left(i,j\right)$ and $I_{0}\left(i,j\right)$ represent the image intensities of certain pixels at the position (i,j) in the current and background frames, respectively. We first stored the image frame that only contained of the static target scene as the background image. ∆I(i,j) was then compared with the input threshold value. The choice of threshold was very important in determining the success of the motion detection. The selection of the threshold in this algorithm was based on Otsu’s binarization method [27]. Finally, if ∆I(i,j) exceeded the threshold value, it meant that there was a smoke motion in the area that was being monitored. The algorithm could be described by the flowchart shown in Figure 2.

The background subtraction technique had the advantages of easy implementation and fast detection, in addition to being able to provide the complete feature data of the target scene. However, this method was vulnerable and particularly sensitive to the variations in the dynamic scenes, including the sudden illumination changes in the background, shadows, and camera shakes [28]. Therefore, it was suggested that the experiment needed to be carried out under stable meteorological conditions. The infrared target array and MWIR sensor also needed to remain immovable during the field trial.

**Figure 3:**
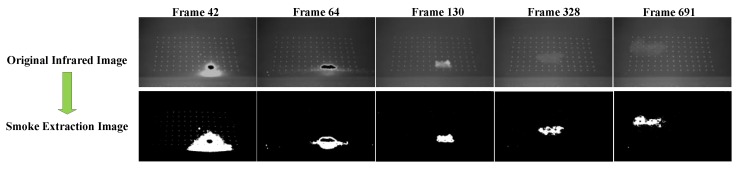
Original infrared image and smoke extraction image at frame 42, 64, 130, 328, and 691. The images in the first line represent the infrared images from the sensor, while the images in the second line represent the images after the background subtraction processing.

Figure 3 presents an example of the smoke motion, using the background subtraction method. The original image and smoke extraction image at frames 42, 64, 130, 328, and 691 are presented. The result proved that the background subtraction could be applied to output the location of the smoke contour practically in real-time.

### 3.2. Transmittance Interpolation

**Figure 4:**
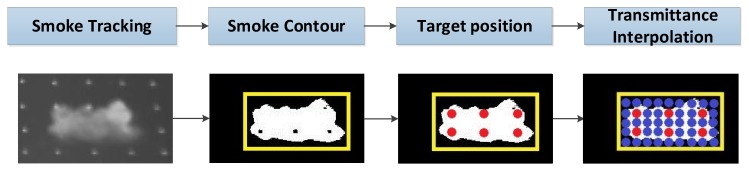
The algorithm flowchart of transmittance interpolation.

Smoke transmittance has been defined as the fraction of the incident electromagnetic radiation, which passes through smoke obscurants [29]. In this section, the smoke transmittance of each pixel within the research area was investigated by the transmittance interpolation. The algorithm flowchart is shown in Figure 4. The motion of the smoke cloud was first tracked, while the smoke contour was drawn by the background subtraction method, as mentioned above. The targets within the smoke contour were then positioned and the smoke transmittance of these targets were calculated as the known data by the grayscale smoke transmittance model [24]. Finally, the transmittance of each pixel within the research area was obtained, based on the transmittance linear interpolation. In the mathematical field of the numerical analysis, interpolation was used to construct additional data points within the range of a discrete set of known values [30]. In the proposed method, an empty two-dimensional matrix, which included the smoke contour, was created. Based on the known smoke transmittance of the study targets, a linear interpolation was applied to fill in the smoke transmittance matrix.

### 3.3. Effective Shielding Area

The effective shielding area of the smoke referred to the area that the smoke cloud could cover when the smoke transmittance met the threshold for shielding [21]. Using the background subtraction and transmittance interpolation methods that were mentioned above, a two-dimensional matrix that contained the smoke transmittance of each pixel was determined. If the smoke transmittance of a certain pixel was more than the input transmittance threshold, the pixel was defined as the effective shielding point. By the accumulation of all of the effective shielding points within the matrix, the total amount of the effective shielding points could be obtained. When combined with the parameters of the thermal infrared imager and field trial, the effective shielding area was calculated using Equation (2), as follows [31]:

(2) $$S=n∆{\left[R\text{×}d/(f\text{×}\cos\alpha )\right]}^{2}$$

where S stands for the effective shielding area, n is the total number of effective shielding points, R refers to the distance between the thermal infrared imager and the smoke explosion site, f is lens focal length d is the pixel spacing, and α is the horizontal observation angle.

In the field trial, the lens focal length, f, and the pixel spacing, d, were constants for the same model of the thermal infrared imager. The distance between the thermal infrared imager and smoke explosion site, R, was chosen according to the spatial resolution of the sensor. α was the horizontal observation angle. With all of these parameters that were obtained above, the effective shielding area of the smoke could finally be accurately calculated.

## 4. Results and Discussion

Using the aforementioned methodology to calculate the effective shielding area of the smoke, a field trial example will be illustrated and discussed in this section.

**Figure 5:**
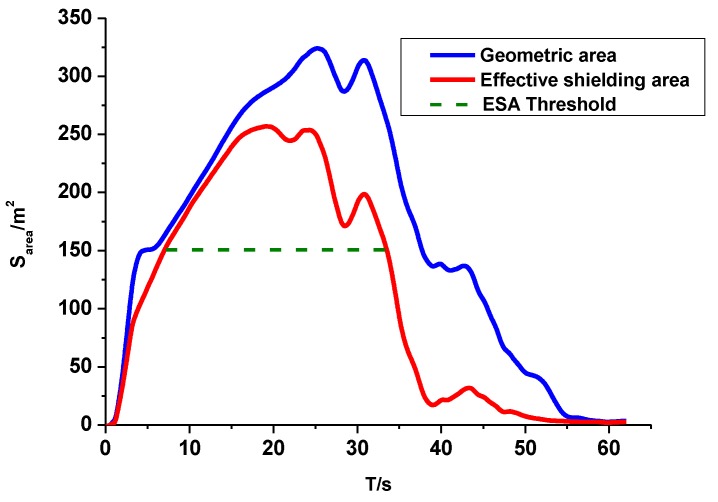
Smoke area curves during the smoke diffusion process. The blue line represents the smoke geometric area, the red line represents effective shielding area of the smoke, and the green dashed line represents the threshold for effective shielding area (ESA) of the smoke.

In the field trial, a smoke ammunition was detonated between the thermal infrared sensor and the target array. The thermal infrared sensor was used to track and record the smoke motion process in the infrared spectra, with wavelengths of 3–5 μm. The results from the frame-by-frame analysis of the images are shown in Figure 5

As shown in Figure 5, the blue line, red line, and green dashed line referred to the smoke geometric area, effective shielding area, and effective shielding area threshold, respectively. At first, a smoke screen with a strong infrared radiation was formed shortly after the detonation of the smoke ammunition. As a result of the effect of the atmospheric turbulence and the increase in the smoke concentration, the smoke screen started to diffuse and enlarged gradually until it reached its maximum shielding extent. After this, the smoke shielding area began to reduce and completely vanished in the end, because of a decrease in the smoke temperature and a decline in the smoke concentration. In Figure 5, the two area curves almost overlapped with each other during the period of 0–15 s. This was caused by the high temperature of the smoke screen at the beginning. Thus, the smoke screen could emit a strong infrared radiation and the geometric area was almost equal to the effective shielding area during this time. From 15 s to 60 s, since the smoke screen started to diffuse, the effectiveness of the smoke in obscuring the area, began to reduce. Therefore, during this period of time, the value of the effective shielding area of the smoke was less than the geometric area.

In addition, the green dashed line in Figure 5 provides a threshold for the effective shielding area. The threshold was an input parameter, which could be adjusted based on the different requirements for the smoke obscuring capability. In this experiment, according to the target size, the threshold for the effective shielding area of the smoke was set as 150 $m^{2}$ in Figure 5, as an example to conduct further analysis. The smoke screen could only provide an adequate shielding area for the potential targets if the effective shielding area was more than the threshold. Based on the effective shielding area of the smoke curve and the input threshold for the effective shielding area of the smoke, the maximum effective shielding area and average effective shielding area could be calculated in order to better evaluate the field infrared smoke shielding performance, as follows.

The maximum effective shielding area of the smoke was the maximum value that was obtained from all of the effective shielding areas. In Figure 5, according to the effective shielding area curve, the maximum was 251 $m^{2}$. The average effective shielding area was the average value for the effective shielding area of the smoke, during the period of time when the effective shielding area met the threshold. In Figure 5, the average effective shielding area is 198 $m^{2}$. The results are shown in Table 1.

According to the effective shielding area of the smoke, which was calculated by the proposed approach in this study, the military researchers could efficiently determine the number of smoke ammunitions and smoke-releasing time on the battlefield, which contributed greatly to the military tactics.

## 5. Conclusions

This paper has investigated a new approach for the effective shielding area of the field infrared smoke. The approach combines the techniques of background subtraction with the transmittance interpolation. The results from the experiment demonstrate that this approach can facilitate the evaluation work of the field infrared smoke screen performance. According to the findings of the study, the conclusions are as follows:

(1) With the help of the thermal infrared sensor and the motion detection technique, the background subtraction can be applied to efficiently display the contour of the smoke cloud in real-time.
(2) Based on the contour of the smoke cloud, a smoke transmittance matrix can be created by the linear interpolation method.
(3) The effective shielding area can be calculated by the accumulation of the effective shielding pixel points in the transmittance matrix and allows for the evaluation of the field smoke shielding performance.

## Figures and Tables

**Table 1:** Area parameters for smoke shielding performance.

Maximum effective shielding area/m^2^	251
Average effective shielding area/m^2^	198
